# Exploring the brain epitranscriptome: perspectives from the NSAS summit

**DOI:** 10.3389/fnins.2023.1291446

**Published:** 2023-10-20

**Authors:** Sung-Min Lee, Bonsang Koo, Clément Carré, André Fischer, Chuan He, Ajeet Kumar, Kathy Liu, Kate D. Meyer, Guo-li Ming, Junmin Peng, Jean-Yves Roignant, Erik Storkebaum, Shuying Sun, Davide De Pietri Tonelli, Yinsheng Wang, Yi-Lan Weng, Luigi Pulvirenti, Yanhong Shi, Ki-Jun Yoon, Hongjun Song

**Affiliations:** ^1^Department of Biological Sciences, Korea Advanced Institute of Science and Technology (KAIST), Daejeon, Republic of Korea; ^2^KAIST Stem Cell Center, Korea Advanced Institute of Science and Technology (KAIST), Daejeon, Republic of Korea; ^3^Transgenerational Epigenetics & Small RNA Biology, Centre National de la Recherche Scientifique, Laboratoire de Biologie du Développement - Institut de Biologie Paris Seine, Sorbonne Université, Paris, France; ^4^Department for Epigenetics and Systems Medicine in Neurodegenerative Diseases, German Center for Neurodegenerative Diseases (DZNE), Göttingen, Germany; ^5^Department of Chemistry, Howard Hughes Medical Institute, The University of Chicago, Chicago, IL, United States; ^6^Department of Biochemistry and Molecular Biology, Howard Hughes Medical Institute, The University of Chicago, Chicago, IL, United States; ^7^Department of Biochemistry and Biophysics, University of Pennsylvania, Philadelphia, PA, United States; ^8^Department of Biochemistry, Duke University School of Medicine, Durham, NC, United States; ^9^Department of Neurobiology, Duke University School of Medicine, Durham, NC, United States; ^10^Department of Neuroscience and Mahoney Institute for Neurosciences, Perelman School of Medicine, University of Pennsylvania, Philadelphia, PA, United States; ^11^Institute for Regenerative Medicine, University of Pennsylvania, Philadelphia, PA, United States; ^12^Department of Cell and Developmental Biology, Perelman School of Medicine, University of Pennsylvania, Philadelphia, PA, United States; ^13^Department of Psychiatry, Perelman School of Medicine, University of Pennsylvania, Philadelphia, PA, United States; ^14^Department of Structural Biology, St. Jude Children's Research Hospital, Danny Thomas Place, Memphis, TN, United States; ^15^Department of Developmental Neurobiology, St. Jude Children's Research Hospital, Danny Thomas Place, Memphis, TN, United States; ^16^Center for Integrative Genomics, Faculty of Biology and Medicine, University of Lausanne, Lausanne, Switzerland; ^17^Institute of Pharmaceutical and Biomedical Sciences, Johannes Gutenberg-University Mainz, Mainz, Staudingerweg, Germany; ^18^Donders Institute for Brain, Cognition and Behaviour and Faculty of Science, Radboud University, Nijmegen, Netherlands; ^19^Department of Physiology and Brain Science Institute, Johns Hopkins University School of Medicine, Baltimore, MD, United States; ^20^Neurobiology of miRNA, Fondazione Istituto Italiano di Tecnologia (IIT), Genova, Italy; ^21^Department of Chemistry, University of California, Riverside, CA, United States; ^22^Department of Neurosurgery, Houston Methodist Neurological Institute, Houston, TX, United States; ^23^Neuroscience School of Advanced Studies, London, United Kingdom; ^24^Department of Neurodegenerative Diseases, Beckman Research Institute of City of Hope, Duarte, CA, United States; ^25^The Epigenetics Institute, Perelman School of Medicine, University of Pennsylvania, Philadelphia, PA, United States

**Keywords:** RNA modifications, epitranscriptome, neuroepitranscriptomics, neurodevelopment, neurogenesis, glioblastoma

## Abstract

Increasing evidence reinforces the essential function of RNA modifications in development and diseases, especially in the nervous system. RNA modifications impact various processes in the brain, including neurodevelopment, neurogenesis, neuroplasticity, learning and memory, neural regeneration, neurodegeneration, and brain tumorigenesis, leading to the emergence of a new field termed neuroepitranscriptomics. Deficiency in machineries modulating RNA modifications has been implicated in a range of brain disorders from microcephaly, intellectual disability, seizures, and psychiatric disorders to brain cancers such as glioblastoma. The inaugural NSAS Challenge Workshop on Brain Epitranscriptomics hosted in Crans-Montana, Switzerland in 2023 assembled a group of experts from the field, to discuss the current state of the field and provide novel translational perspectives. A summary of the discussions at the workshop is presented here to simulate broader engagement from the general neuroscience field.

## Introduction

**Figure 1:**
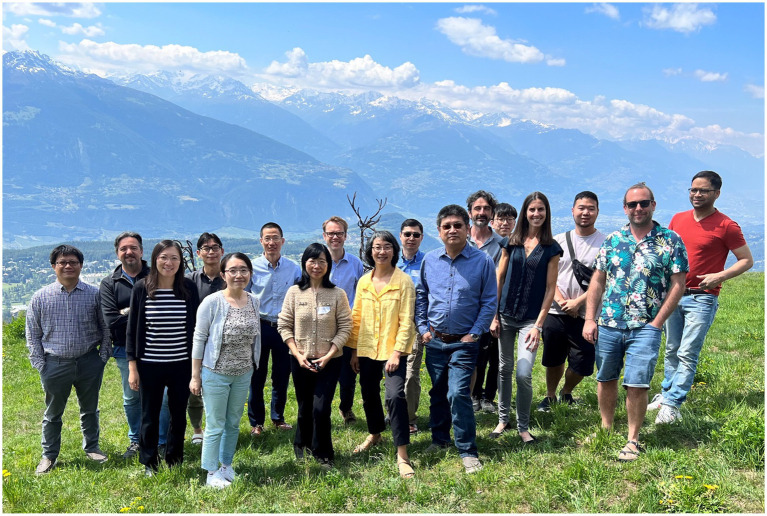
Participants of the NSAS challenge workshop on brain epitranscriptomics at Crans-Montana, Switzerland. From left to right, Yi-Lan Weng, Davide De Pietri Tonelli, Shuying Sun, Ki-Jun Yoon, Kathy Liu, Yinsheng Wang, Yanhong Shi, Erik Storkebaum, Guo-li Ming, Junmin Peng, Hongjun Song, Jean-Yves Roignant, Sung-Min Lee, Kate D. Meyer, Bonsang Koo, Clément Carré, and Ajeet Kumar.

Hundreds of RNA modifications, which occur in mRNA, tRNA, and rRNA, exert control over almost every facet of RNA metabolism, including splicing, export, stability, and translation (Livneh et al., 2020; Salinas et al., 2020). Recent studies have provided emerging evidence highlighting the essential functions of RNA modifications in numerous biological processes within the nervous system. These processes range from neurogenesis, development, plasticity, learning and memory, to regeneration and degeneration, as well as brain tumors, thus giving rise to the new field of neuroepitranscriptomics. Various genes associated with RNA modification pathways have been implicated in a range of brain disorders, spanning from microcephaly, intellectual disability, seizures, and psychiatric disorders, to brain cancers. Hosted at the Neuroscience School of Advanced Studies (NSAS) and directed by Hongjun Song (University of Pennsylvania, United States), the NSAS Challenge Workshop on Brain Epitranscriptomics in Crans-Montana, Switzerland (June 12th–16th, 2023), assembled a group of experts from the field, with the primary objective of offering valuable insights into the current state of the field and delving into potential novel translational perspectives (Figure 1). Here we provide a summary of the discussions at the workshop to encourage broader engagement with the general neuroscience field.

## Epitranscriptomic regulation of the nervous system development

**Figure 2:**
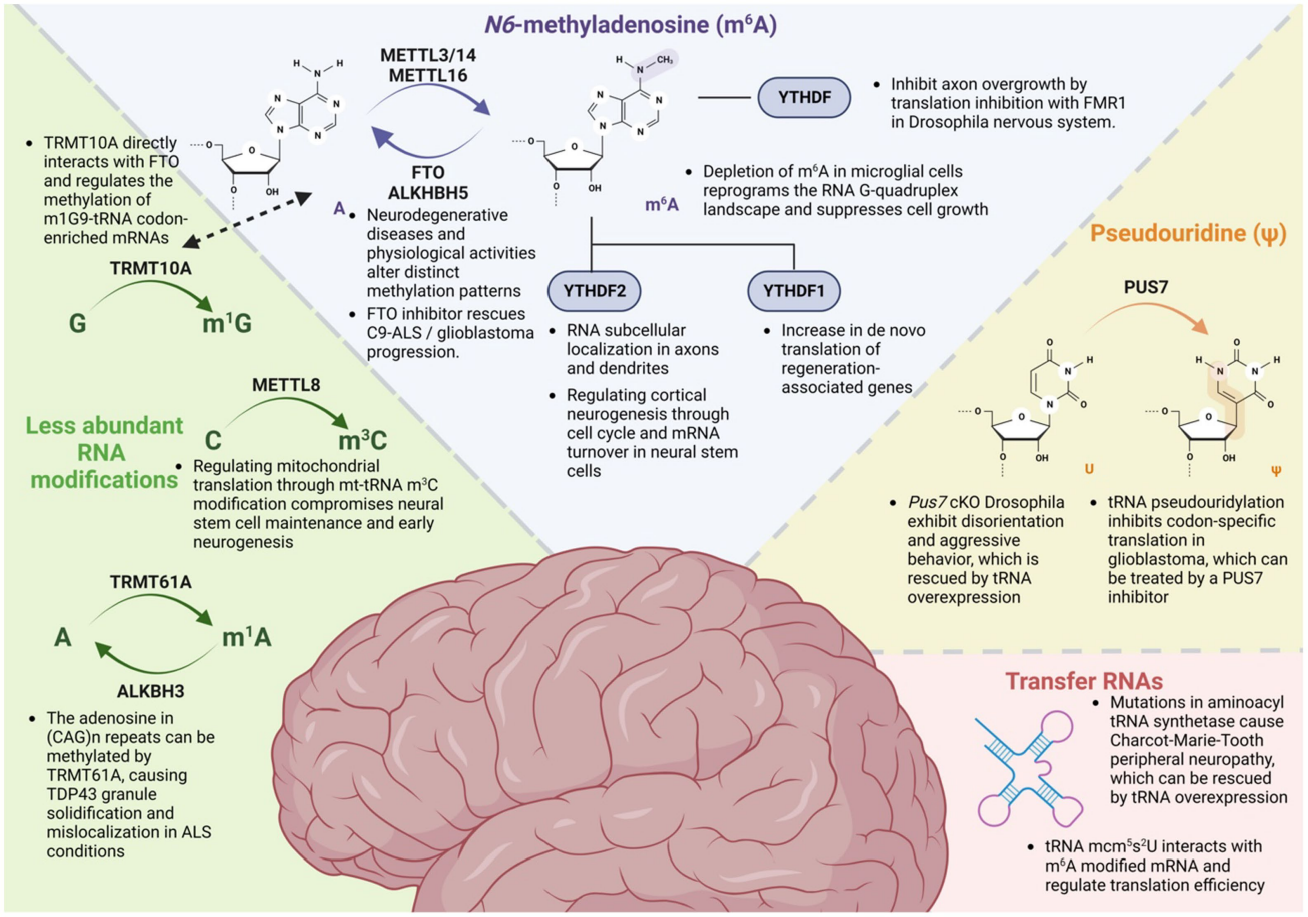
Epitranscriptomic modifications and their roles in the nervous system. Multiple RNA modifications play a crucial role in nervous system development, regeneration, and neurodegenerative disorders. Among these modifications, N6-methyladenosine (m^6^A) regulates RNA subcellular localization, turnover, and translation with their reader proteins. Another important modification, pseudouridine (ψ), known as the second most abundant RNA modification, modulates behavior patterns and influences glioblastoma through a tRNA-dependent mechanism. Additionally, tRNA exhibits various modifications and has been found to be correlated with neurological disorders, such as Charcot–Marie–Tooth disease. Furthermore, recent findings indicate a clear correlation between the nervous system and emerging RNA modifications, including 1-methylguanosine (m^1^G), 3-methylcytidine (m^3^C), and N1-methyladenosine (m^1^A).

Recent studies suggest that epitranscriptomic RNA modification plays a crucial role in the development of the nervous system (Vissers et al., 2020). These modifications involve various processes, such as neural stem cell regulation, axonal projections, dendritic development, and synapse formation (Angelova et al., 2018; Park et al., 2020; Figure 2). Jean-Yves Roignant (University of Lausanne, Switzerland) commenced the meeting by introducing the fundamental concept of epitranscriptomics, focusing on the most abundant internal mRNA modification, *N^6^*-methyladenosine (m^6^A), and its functions in the nervous system. He presented evidence demonstrating that depletion of m^6^A and its reader protein Ythdf in *Drosophila* led to axonal overgrowth at the neuromuscular junction and in mushroom bodies (Worpenberg et al., 2021). Ythdf was found to recruit Fmr1, which inhibits the translation of 5’ UTR m^6^A-tagged mRNA such as *futsch*, which encodes a microtubule associated protein, thereby regulating proper axon development.

Guo-li Ming (University of Pennsylvania, United States) first discussed the role of m^6^A modification in regulating cortical neurogenesis in mice and human induced pluripotent stem cell (iPSC)-derived forebrain organoids via cell cycle and mRNA decay regulation (Yoon et al., 2017). She then discussed her recent work on the m^3^C modification of mt-tRNA^Thr/Ser^, which is mediated by Mettl8 in cortical neural stem cells in mice and humans (Zhang F. et al., 2023). Depletion of Mettl8 resulted in decreased m^3^C tRNA modifications and impaired mitochondrial protein translation, which in turn compromises neural stem cell maintenance, leading to reduced neurogenesis of superficial layer neurons in mice.

Ki-Jun Yoon (Korea Advanced Institute of Science and Technology, South Korea) presented findings indicating that m^6^A modification has minimal effect on mRNA stability in differentiating postmitotic neurons. Instead, it was observed that m^6^A modification promotes the distal localization of mRNAs associated with cytoskeletal rearrangement and cell adhesion. As a result, depletion of Mettl14 or Ythdf2 led to impaired axonal projections and growth cone advancement in the developing mouse cortex. Additionally, Kate Meyer (Duke University, United States) presented data that showcased the consequences of genetic loss of m^6^A or reader proteins, such as YTHDF2 or YTHDF3, on the altered subcellular localization of mRNAs within the neurites of mouse hippocampal neurons (Flamand and Meyer, 2022).

Collectively, these findings underscore the diverse functions and mechanisms of epitranscriptomic regulation during the development of the nervous system, from flies to mammals.

## Epitranscriptomic modulation of regeneration and neurodegeneration

Researchers are increasingly exploring the role of epitranscriptomic modifications in the development and progression of various neurodegenerative diseases, such as Alzheimer’s disease (AD), Parkinson’s disease, and amyotrophic lateral sclerosis (ALS; Angelova et al., 2018; Park et al., 2020). It has been suggested that dysregulated regeneration and axon damage are implicated in the pathogenesis of neurodegenerative diseases (Qian and Zhou, 2020; Wu et al., 2023). Hongjun Song (University of Pennsylvania, United States) introduced the roles of RNA m^6^A modification during mouse peripheral nervous system (PNS) nerve injury (Weng et al., 2018). In this system, m^6^A modifications positively regulate the regeneration of damaged axons by promoting the protein translation of regeneration-associated genes that were tagged by m^6^A. The loss of m^6^A and YTHDF1 resulted in reduced axon regeneration and decreased *de novo* protein synthesis induced by injury in mice.

AD is a prevalent neurodegenerative condition that affects older individuals and is characterized by the development of dementia and cognitive impairment. Andre Fischer (DZNE & University of Göttingen, Germany) presented findings demonstrating that m^6^A hypomethylation in mouse brains of AD models led to a reduction in local translation within synapses, resulting in impaired neuronal plasticity (Castro-Hernández et al., 2023). Although general transcriptomic and translational regulation remained unchanged, there was a decrease in CAMK2 translation levels, specifically within synapses in these models. Conversely, regular exercise, used to model activity-dependent mechanisms, induced expression of the m^6^A demethylase FTO, which led to m^6^A hypomethylation in the 5’ UTR of mRNAs and an upregulation of synaptic proteins. In neurodegenerative diseases and physiological conditions, a comparable mechanism of hypomethylation can produce contrasting outcomes depending on the specificity of m^6^A residues and the mediators involved.

Yinsheng Wang (University of California Riverside, United States) discussed how adenosine in the CAG repeat RNA can be m^1^A methylated by TRMT61A and demethylated by ALKBH3. The m^1^A modification in the CAG repeat RNA can bind to TDP-43 and result in its truncation, cytoplasmic redistribution, and aggregation, which recapitulated TDP-43 proteinopathy observed in neurodegenerative diseases, including ALS. Shuying Sun (Johns Hopkins University, United States) highlighted the m^6^A dysregulation in C9ORF72 repeat expansion-linked ALS and frontotemporal dementia (FTD; Li et al., 2023). Both iPSC-differentiated motor neurons and multiple postmortem brain regions from patients with C9-ALS/FTD exhibited reduced expression of Mettl3 and Mettl14, leading to hypomethylated transcripts with extended half-lives. Furthermore, the presence of m^6^A peaks in the intronic region surrounding the repeat expansion of C9ORF72 regulates the repeat RNA stability. Loss of m^6^A can increase the accumulation of toxic repeat RNAs and poly-dipeptides. The disease phenotypes could be ameliorated by elevating m^6^A levels, such as through increasing the methyltransferase METTL14 or reducing the demethylase FTO, thereby restoring the overall m^6^A level on transcripts and decreasing the neurotoxicity.

Junmin Peng (St. Jude Children’s Research Hospital, United States) demonstrated the use of the latest TMT-LC/LC–MS/MS technology in analyzing brain proteomics samples from a mouse model of AD and hundreds of AD patients. This approach led to the discovery of a large set of novel protein components of amyloid plaque, including Aβ-correlated proteins and numerous RNA-binding proteins (Bai et al., 2020; Zaman et al., 2023). His laboratory further established a new mouse model with induced aberrant splicing and crossing this mouse model with the commonly used 5xFAD model more accurately replicated human AD pathology (Chen et al., 2022). By employing a meticulous approach to analyze different layers of proteomes, such as the whole proteome, aggregated proteome, and protein modifications, it is now possible to investigate over 10,000 proteins and their associated post-translational modifications in AD patients. Interestingly, in multi-omics analysis encompassing both transcriptome and proteome data, the consistency between translational regulation and transcriptomic regulation was found to be approximately 50% for accumulated proteins, suggesting that post-transcriptional regulatory mechanisms can significantly impact overall gene expression (Bai et al., 2020). Additionally, the role of RNA modifications was also implicated in the brains of human AD patients, where several RNA methyltransferase proteins, such as METTL3, METTL7A, and METTL7B were found to be increased in AD brains from a meta-analysis (Bai et al., 2021).

## Advances in m^6^A detection techniques

Conventional antibody-based RNA modification detection methods have limitations in terms of antibody specificity and resolution. Chuan He (University of Chicago, United States) discussed the use of chemical-based RNA modification sequencing techniques, such as m^6^A selective allyl chemical labeling (SAC; Hu et al., 2022; Ge et al., 2023), eTAM-seq (Xiao et al., 2023), and pseudouridine bisulfite-induced deletion (BID) sequencing (Dai et al., 2023; Zhang L.-S. et al., 2023). BID-seq has proven effective in uncovering consensus sequences, potential writer proteins, and the functional role of pseudouridylation in stabilizing transcripts. Additionally, BID-seq can detect tissue-specific pseudouridine modifications, including those occurring in stop codon regions.

Kate Meyer utilized deamination adjacent to RNA modification targets (DART) sequencing in neural cells. Unlike antibody-based approaches that require large amounts of RNA, DART-seq can be applied to small samples, even at the single-cell level. Interestingly, m^6^A modifications exhibit heterogeneity across cells, both in terms of the proportion of cells in which a particular RNA is methylated as well as the abundance of m^6^A at individual sites. In addition, mRNAs contain many more sites than previously thought; however, most of these sites occur relatively rarely within individual cells of a population. This argues for the importance of regions of methylation within RNAs as opposed to individual sites. She also introduced a new DART transgenic mouse line that expresses the APOBEC1-YTH protein, enabling *in vivo* single-cell mapping of m^6^A in the brain and other tissues of interest. *In vivo* single-cell DART-seq in the mouse cortex identified over 170,000 m^6^A sites across different cell types, and revealed that microglial cells have substantial hypomethylation of their mRNAs compared to other cell types in the cortex. Lastly, she presented a new technology which combines the APOBEC1-YTH system with a fluorescent reporter mRNA to create a genetically encoded m^6^A sensor capable of detecting m^6^A dynamics in live cells.

## Epitranscriptomic role in functional RNAs

Although the majority of epitranscriptomic studies discussed in this meeting have focused on mRNAs, the potential role of RNA modifications in different types of RNAs was also discussed. Jean-Yves Roignant pointed out the potential importance of m^5^C and pseudouridine, which are installed by NSUN6 and PUS7, respectively, on intellectual disability and aggressive behavior, utilizing multiomics analysis. In particular, the lack of PUS7 in *Drosophila* led to aggressive behavior and metabolic changes, which were rescued by overexpressing tRNA^Asp^. It has been suggested that epitranscriptomic dysregulation of metabolism may contribute to mental disorders (de Brouwer et al., 2018).

Davide De Pietri Tonelli (Italian Institute of Technology-Genova, Italy) explored the involvement of small noncoding RNAs, such as the Piwi-interacting RNAs (piRNAs), in adult neurogenesis (Gasperini et al., 2023). Depletion of Piwil2 and piRNAs through knockdown or the aging process results in an increased activation of the ROS pathway and inflammation in neural stem cells of the adult mouse hippocampus, ultimately leading to increased astrogenesis. He also discussed the possibility of epitranscriptomic modifications on piRNAs and their functional roles.

Kathy Fange Liu (University of Pennsylvania, United States) proposed the concept of direct and indirect coordination of RNA modifications between mRNA and other RNAs. She highlighted the interaction between TRMT10A, the tRNA m^1^G methyltransferase, and FTO, which influences the m^6^A methylation levels of a subset of messenger RNAs (Ontiveros et al., 2020). Interestingly, these target transcripts exhibited an enrichment of m^1^G9-tRNA codons, suggesting the presence of coordinated mRNA and tRNA methylation. Additionally, she discussed the direct interaction between m^6^A-modified mRNA and tRNA 5-methoxycarbonylmethyl-2-thiouridine (mcm^5^s^2^U), which synergistically promotes translation efficiency.

## From epitranscriptome regulation to clinical translation

Yi-Lan Weng (Houston Methodist Research Institute, United States) discussed the impact of m^6^A deficiency in human microglial cells. He highlighted that m^6^A deficiency leads to the repression of cell growth and the activation of a viral mimicry response through reprogramming of the RNA G-quadruplexes (rG4) landscape. The increased presence of rG4 structures promoted ZBP1 dimerization, which in turn resulted in cell death.

Erik Storkebaum (Donders Institute and Radboud University, Nijmegen, Netherlands) discussed the molecular mechanisms underlying two neurodegenerative diseases and their clinical implications. Firstly, he described how ALS-associated mutations in the nuclear localization signal (NLS) of the FUS protein are intrinsically toxic to both motor neurons and skeletal muscles, leading to structural and functional neuromuscular junction defects and motor neuron degeneration (Scekic-Zahirovic et al., 2016, 2017). At the molecular level, FUS collaborates with the ETS-transcription factor Erm/Etv5 to stimulate transcription of acetylcholine receptor subunit genes. Consequently, FUS NLS mutations result in reduced expression of acetylcholine receptor subunit genes in subsynaptic myonuclei, leading to reduced neuromuscular endplate size and neuromuscular transmission defects in an FUS^ΔNLS^ mouse model, similar to the electrophysiological defects in myasthenic syndromes (Picchiarelli et al., 2019). This suggests that drugs currently used to treat myasthenic syndromes may possibly be repurposed to help alleviate neuromuscular dysfunction in *FUS*-ALS. Secondly, he discussed Charcot–Marie-Tooth peripheral neuropathy (CMT), where mutations in glycyl-and tyrosyl-tRNA synthetases (GlyRS and TyrRS) inhibit mRNA translation, leading to peripheral neuropathy phenotypes (Niehues et al., 2015). Mechanistically, CMT-mutant GlyRS proteins are still able to bind tRNA^Gly^ but fail to release it. This tRNA^Gly^ sequestration depletes the cellular pool of tRNA^Gly^, leaving insufficient tRNA^Gly^ for wild type GlyRS (in heterozygous CMT-GlyRS patients). This results in insufficient production and supply of glycyl-tRNA^Gly^ to the ribosome and stalling of the ribosome on glycine codons (Zuko et al., 2021). Ribosome stalling subsequently activates the integrated stress response through the eIF2α kinase GCN2, selectively in disease-affected motor and sensory neurons (Spaulding et al., 2021). The translation defect and peripheral neuropathy phenotypes could be rescued by overexpressing tRNA^Gly^, suggesting a novel mechanism-based therapeutic approach (Zuko et al., 2021).

Last, Yanhong Shi (City of Hope, United States) presented two distinct molecular mechanisms involved in glioblastoma (GBM) progression, glioblastoma stem cell (GSC) self-renewal, and potential clinical approaches to target GBM using inhibitors of RNA modification machinery. She discussed the role of m^6^A modification in GSC self-renewal and GBM progression. m^6^A methylation in GSCs is catalyzed by METTL3 and METTL14 proteins. Dysregulation of these proteins leads to hypomethylation of transcripts in GBM, promoting the growth and self-renewal of GSCs. Treatment with an FTO inhibitor was shown to increase m^6^A levels in GSCs that resulted in reduced GSC self-renewal and suppressed GBM progression, suggesting that targeting the m^6^A machinery can be a viable approach to treat GBM (Cui et al., 2017). She also highlighted the role of PUS7 in GSC self-renewal and GBM progression through pseudouridylation of tRNA, which inhibits codon-specific translation in GSCs. This mechanism is involved in regulating GSC growth and self-renewal via the TYK2-mediated IFN pathway. Moreover, her laboratory identified PUS7 inhibitors using a structure-based virtual screening coupled with *in vitro* enzymatic activity screening. The inhibitors were able to reduce pseudouridine levels and suppress GBM tumorigenesis (Cui et al., 2021). These results suggest that inhibitors of RNA modification machineries can be used as therapeutic candidates for targeting GBM and other cancers (Cerneckis et al., 2022).

## Exploring innovative therapeutic approaches beyond current technical limitations

**Figure 3:**
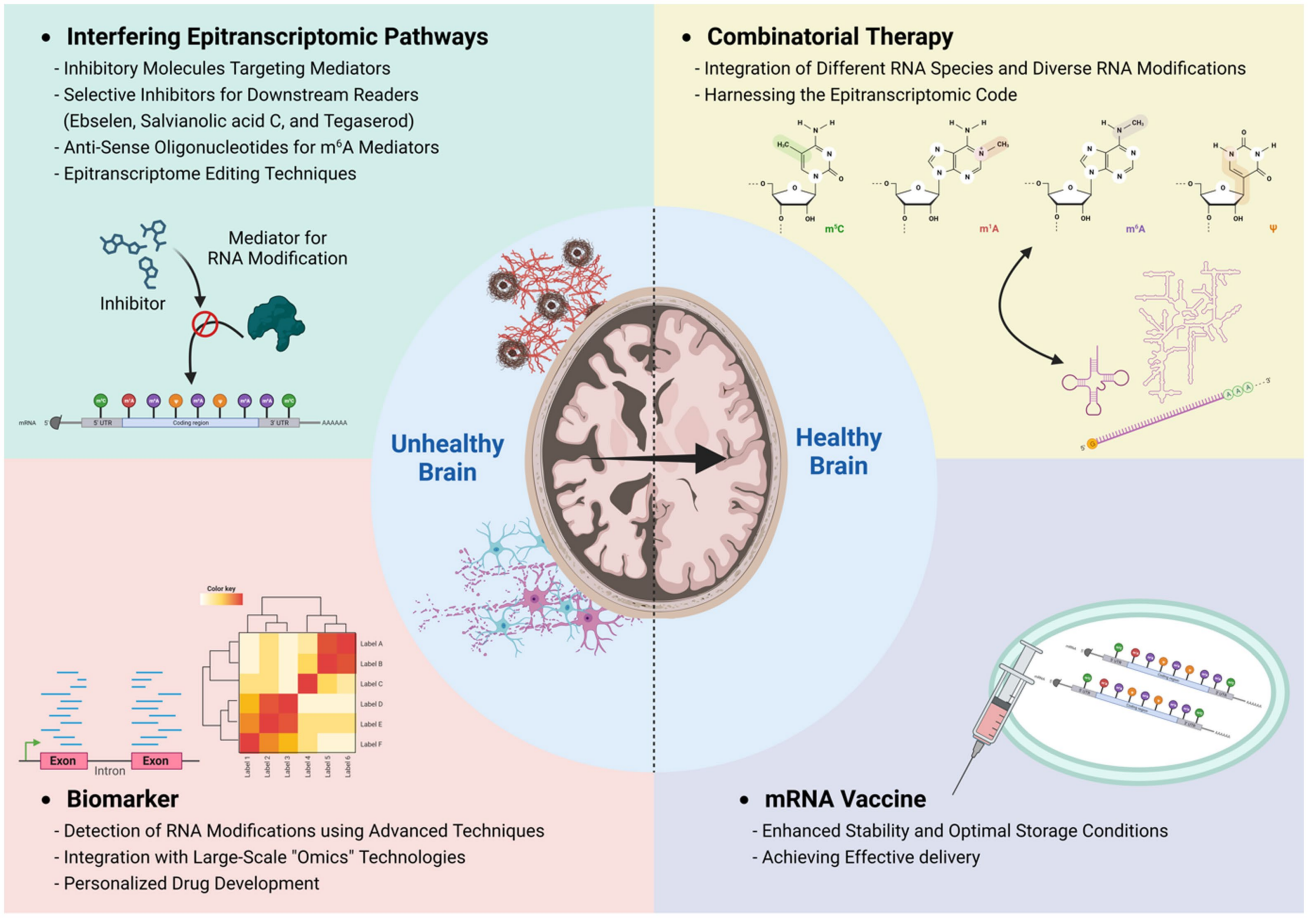
Epitranscriptomic approach to therapeutic applications. Investigating epitranscriptome alterations in pathological conditions through advanced detection techniques, including nanopore sequencing, will facilitate the identification of novel biomarkers specific to various neuropathologies. Furthermore, the integration of a wide array of epitranscriptomic RNA modifications across various RNA species offers the prospect of formulating combinatorial therapies and bolstering the efficacy of mRNA vaccines, paving the way toward personalized medicine approaches for improved patient outcomes.

The workshop participants had an active discussion of the future translational potential of neuroepitranscriptomic research and highlighted a few areas (Figure 3).

### Epitranscriptomic dysregulation as a biomarker for brain disorders

Epitranscriptomic dysregulation has been found in a number of brain disorders, raising the possibility of its use as a biomarker for diagnosis and treatment efficacy of these disorders. Currently, traditional methods of detecting RNA modifications have limitations in terms of resolution, quantitative analysis with stoichiometry, available material quantity, and large sample requirements. However, recent advances in the field have introduced novel methods that use fusion proteins and chemical-based sequencing, allowing for analysis at the single base pair and single-cell level (Zhang et al., 2019; Hu et al., 2022; Tegowski et al., 2022; Dai et al., 2023; Shu et al., 2023; Xiao et al., 2023). Furthermore, direct nanopore sequencing coupled with postprocessing machine learning is expected to become a potential platform for identifying various RNA modifications on individual transcripts (Leger et al., 2021; Jain et al., 2022; Stephenson et al., 2022). Combining RNA modification analysis with other large-scale “omics” technologies enables us to comprehensively investigate the interplay between RNA modifications and the proteome, epigenome, and transcriptome to uncover how these multiple regulatory pathways influence each other. Coupling these strategies with single-cell approaches will even further propel our understanding of these processes in diverse cell types.

When dealing with the diagnosis, prognosis, and treatment of diseases related to RNA modifications, embracing a comprehensive approach that encompasses various mechanisms from different RNA modifications (on different RNA molecules) has potential for novel approaches to personalized medicine. By deciphering the regulation of gene expression through RNA modifications and identifying biomarkers that arise from perturbations of the epitranscriptome in pathological conditions (e.g., tRNA fragmentation), we can develop customized drugs tailored to individual patients and identify specific RNA biomarkers for diverse diseases.

To unravel the intricate “epitranscriptome” as an additional layer of the biological system, a future large-scale human cohort study is indispensable for comprehensive exploration. Additionally, the use of disease models such human NPCs (Brazane et al., 2023) and iPSC-derived organoid systems, in combination with mouse and other models with loss-of-function for the writers/erasers/readers of various RNA modifications, will provide unprecedented insights into the roles of RNA modifications in brain disease.

### Interfering with epitranscriptomic pathways for therapeutic applications

To translate novel findings in epitranscriptomic gene regulation to human disease treatment, researchers have developed specific molecules aimed at inhibiting epitranscriptomic modulators. Currently, several therapeutic strategies have been proposed involving m^6^A demethylase inhibitors for various diseases, including glioblastoma and neurodegenerative conditions (Selberg et al., 2021; Huff et al., 2022; You et al., 2022). However, due to the diverse effects of m^6^A modification on RNA metabolism, generally manipulating m^6^A levels could result in potential side effects. As a solution, researchers have recently developed inhibitors that target downstream reader proteins of the m^6^A pathway. An example of such an inhibitor is Ebselen, which binds to the hydrophobic pockets in the YTH domain of YTHDF1 and YTHDF2 (Micaelli et al., 2022).

The potential redundant functions of YTHDF proteins pose a challenge when attempting to target a specific reader protein to a disease mechanism. However, the variations in protein structures among YTHDF proteins also indicate the potential for developing selective inhibitors that target each reader protein individually (Sikorski et al., 2023). So far, two compounds, salvianolic acid C (Zou et al., 2022) and tegaserod (Hong et al., 2023), have been developed to target YTHDF1. Additionally, several compounds have been designed to specifically target the m^6^A-binding pocket of YTHDF2 (Nai et al., 2022). Moreover, researchers have developed inhibitors for other reader proteins, such as YTHDC1, and these have been tested for specific types of diseases (Li et al., 2022). Furthermore, the application of anti-sense oligonucleotides (ASOs) targeting m^6^A mediators shows promise. Given the structural similarity of reader proteins, ASOs operate in a nucleotide-specific manner, offering improved specificity and enabling more precise treatment of diseases (Kulkarni et al., 2021). Additionally, epitranscriptome editing techniques, such as using a fusion protein of dCas13-APOBEC (Huang et al., 2020), or dCasRx-conjugated methyltransferase or demethylase systems (Xia et al., 2021), can be proposed as a way to specifically control abnormal gene expression responsible for causing diseases. However, for the successful implementation of these systems in a precise and controlled manner that includes cell-type-specific and gene-specific approaches, a better understanding of epitranscriptomic regulation in neurobiology is essential in the future.

### Combinatorial therapy by targeting multiple pathways

This workshop emphasized the importance of crosstalk between various epitranscriptomic elements. While the RNA modification m^6^A has been extensively studied over the past decade, it is crucial to acknowledge the existence of other elements, such as rare RNA modifications, RNA repeats, and structural formations that may exhibit specific interactions with their modulators. Furthermore, RNA modification on snRNA, tRNA, and rRNA has been shown to regulate biological systems both directly and indirectly (Dimitrova et al., 2019). Therefore, exploring the epitranscriptomic code, similar to the epigenetic code, is essential for gaining a deeper understanding of human diseases and developing effective treatments. In this context, inhibitors targeting different RNA modifications, such as FTO or PUS7 inhibitors, have demonstrated promising results in independently suppressing glioblastoma and reducing self-renewal (Cui et al., 2017, 2021). This suggests the potential of controlling shared disease mechanisms through distinct epitranscriptomic pathways, emphasizing the significance of a holistic approach tackling multiple epitranscriptomic pathways that might offer better outcomes. Considering the interactions between different RNA modifications, the reconciliation of diverse regulatory pathways can potentially overcome the current limitations of inhibitors, leading to more precise and effective medications. However, it is crucial to acknowledge that a combinatorial approach involving different epitranscriptomic pathways may influence each other (Ontiveros et al., 2020) and could potentially lead to unexpected side effects, necessitating further study for combinatorial therapies.

### mRNA-based vaccines adopting RNA modifications for advanced therapeutic approaches

The utilization of mRNA vaccines and drugs offers a promising avenue to stimulate adaptive immunity for targeting infectious diseases (Mei and Wang, 2023). For instance, in the case of COVID-19, incorporating *N^1^*-methylpseudouridine (m^1^Ψ) in mRNA vaccines enhances their stability and translation, reduces the natural immune response to uracil, and facilitates more efficient transportation and storage of the vaccines (Nance and Meier, 2021). Because RNA modifications can regulate various aspects of RNA metabolism, including RNA degradation, splicing, and translation efficiency, incorporating modifications on mRNA vaccines can make mRNA vaccines either antagonistic or synergistic. By adopting an evolutionary-developed viral strategy to increase viral genome stability and replication, potential applications can be explored. For example, through the screening of viral elements, it was revealed that the K5 element from *Kobuvirus* enhances mRNA stability by interacting with ZCCHC2 and TENT4 proteins to form a mixed tailing, which is elongated with mixed sequences, thereby delaying deadenylation (Seo et al., 2023). By strategically manipulating different RNA modifications and their respective residues in mRNA vaccines, it becomes feasible to optimize drug efficacy and enable intricate and personalized therapies for specific diseases.

## Summary

This workshop explored innovative approaches in neuroepitranscriptomic research, highlighting the potential of RNA modifications as biomarkers, and in disease modeling and therapies. Recent advances in high-resolution RNA modification analysis, coupled with integration into other “omics” disciplines, illuminate prospects for personalized medicine. Therapeutically, attention is directed toward inhibiting downstream reader proteins of RNA modification for better specificity and employing combinatorial therapies to enhance treatment outcomes. With the ongoing progression of the field, adopting a multidisciplinary strategy becomes imperative to effectively leverage these breakthroughs. In addition, collaboration between research and clinical communities will play a pivotal role in translating these concepts into tangible benefits for patients.

## Data availability statement

The original contributions presented in the study are included in the article/supplementary material, further inquiries can be directed to the corresponding authors.

## Author contributions

S-ML: Writing – original draft. BK: Writing – original draft. CC: Writing – review & editing. AF: Writing – review & editing. CH: Writing – review & editing. AK: Writing – review & editing. KL: Writing – review & editing. KM: Writing – review & editing. G-lM: Writing – review & editing. JP: Writing – review & editing. J-YR: Writing – review & editing. ES: Writing – review & editing. SS: Writing – review & editing. DD: Writing – review & editing. YW: Writing – review & editing. Y-LW: Writing – review & editing. LP: Writing – review & editing. YS: Writing – review & editing. K-JY: Writing – review & editing. HS: Writing – review & editing.
